# Localization, quantization and interaction with host factors of endogenous HTLV-1 HBZ protein in infected cells and ATL

**DOI:** 10.1186/1742-4690-12-S1-P43

**Published:** 2015-08-28

**Authors:** Goutham U Raval, Carlo Bidoia, Greta Forlani, Giovanna Tosi, Antoine Gessain, Roberto S Accolla

**Affiliations:** 1 Department of Surgical and Morphological Sciences, University of Insubria, Varese, Italy; 2 Departement de Virologie, Institut Pasteur, Paris 75015, France

## 

Human T cell Lymphotropic Virus type 1 (HTLV-1 ) is the etiological agent of a severe form of neoplasia designated Adult T cell Leukemia/Lymphoma (ATLL) It is widely accepted that the viral transactivator Tax-1 is the major viral product involved in the onset but not in the maintenance of neoplastic phenotype as only 30-40% of ATLL cells express Tax-1. It has been recently demonstrated that HBZ (HTLV-1 bZIP factor), a protein encoded by the minus strand of HTLV-1 genome, constantly expressed in infected cells and in ATLL tumor cells, is also involved in the pathogenesis of leukemia. The full role played by HBZ in oncogenesis is still to be explored in detail mainly owing to the unavailability of tools to assess quantitative expression, subcellular location and interaction of HBZ with host factors in ATLL. By the use of the first reported monoclonal antibody against HBZ, 4D4-F3, generated in our laboratory it has been possible to carefully assess for the first time the above parameters in HTLV-1 chronically infected cells and, most importantly, in leukemic cells from patients. Endogenous HBZ is expressed in speckle-like structures localized in the nucleus. The calculated number of endogenous HBZ molecules varies between18.000 to 36.000 molecules per cell, 22-44 fold less than the amount expressed in HBZ transfected cells used by most investigators to assess the expression, function and subcellular localization of the viral protein. HBZ interacts in vivo with p300 and JunD and co-localizes only partially, and depending on the amount of expressed HBZ, not only with p300 and JunD but also with CBP and CREB2.

The possibility to study endogenous HBZ in detail may significantly contribute to a better delineation of the role and presence of HBZ during HTLV-1 infection and cellular transformation.

